# Electron Spin Resonance Dosimetry Studies of Irradiated Sulfite Salts

**DOI:** 10.3390/molecules27207047

**Published:** 2022-10-19

**Authors:** Amanda Burg Rech, Angela Kinoshita, Paulo Marcos Donate, Otaciro Rangel Nascimento, Oswaldo Baffa

**Affiliations:** 1 Departamento de Física, Faculdade de Filosofia Ciências e Letras de Ribeirão Preto, Universidade de São Paulo, Ribeirão Preto 14040-900, SP, Brazil; 2 Pró-Reitoria de Pesquisa e Pós-Graduação, Universidade do Oeste Paulista, Presidente Prudente 19067-175, SP, Brazil; 3 Departamento de Química, Faculdade de Filosofia Ciências e Letras de Ribeirão Preto, Universidade de São Paulo, Ribeirão Preto 14040-900, SP, Brazil; 4 Departamento de Física Interdisciplinar, Instituto de Física de São Carlos, Universidade de São Paulo, São Carlos 13566-590, SP, Brazil

**Keywords:** radiation dosimetry, sulfite, radiation accidents, retrospective dosimetry

## Abstract

The study of new materials for radiation dosimetry is important to improve the present state of the art and to help in cases of accidents for retrospective dosimetry. Sulfites are compounds that contain a sulfur ion, widely used in the food industry. Due to the significant application of these compounds, sulfites are interesting candidates for accidental dosimetry, as fortuitous radiation detectors. The presence of the SO_3_^−^ anion enables its detection by electron spin resonance (ESR) spectroscopy. The Dose–Response behavior, signal stability and other spectral features were investigated for sodium sulfite, sodium bisulfite, sodium metabisulfite and potassium metabisulfite, all in crystalline forms. The ESR spectrum of salts presented stability and proportional response with dose, presenting potential for dosimetry applications.

## 1. Introduction

Sulfites are chemical compounds widely used in the food industry as a preservative and additive. They are sources of SO_2_, which is an antimicrobial agent known since antiquity [1]. 

Parts of sulfites are the bisulfites (HSO_3_^−^) and metabisulfites (S_2_O_5_^2−^), in which the anions SO_3_^2−^, HSO^3−^ and S_2_O_5_^2−^ [2] are present and detectable by electron spin resonance (ESR) spectroscopy, what makes them potential materials to compose dosimeters. Because they are easily found in different applications and are in widespread use, they are a candidate for radiation dosimetry in cases of accidents, in pure form at the location. They can also be employed as a component of a dosimeter or blended with a binder, as is done with alanine, for some specific applications. 

The SO_2_^−^ and SO_3_^−^ anions are commonly cited in ESR papers about speleothems, calcified tissues of shells, snails and corals, because they are generated by ionizing radiation. They are present in the structure of calcites and aragonites as impurities. In these materials, they are characterized by having stability, allowing their use to differ between irradiated from non-irradiated material [3] and other applications such as dosimetry and dating [4,5,6,7,8].

Other inorganic compounds containing the SO_3_**·**^−^ radical ion have already been studied as ESR dosimeters. Bogushevich and Ugolev [9] have discussed some aspects of inorganic ESR dosimeters for medical radiotherapy and have shown that alkaline earth dithionates (S_2_O_6_^2−^) have great potential. Irradiated dithionates exhibit a narrow line stable at room temperature attributed to SO_3_**·^−^** radical anions [10,11]. The oxidation of dithionates (S_2_O_6_^2−^) could result in sulfites (SO_3_^2−^) and dithionites (S_2_O_4_^2−^) [12], also detectable by ESR spectroscopy.

In this work, the spectroscopic characterization of the irradiated sulfites, the behavior of the signal as a function of microwave power, the stability of the signal and the properties of the ESR spectrum of irradiated sulfites as a function of dose of radiation are presented, showing that these compounds have the potential for dosimetric applications. Thus, the goal of this paper is twofold; first, to offer a characterization of the radicals created by ionizing radiation produced by X-ray sources in these compounds by simulating the ESR spectra, and to present the possibility of using these materials as fortuitous dosimeters. To achieve this possible emergency application, the dose response was studied for doses below 20 Gy. In this scenario, materials present at the local site of a radioactive accident that produces stable radicals can be used as a dosimeter giving valuable information to manage the situation. 

## 2. Results and Discussion

### 2.1. ESR Spectra Characterization

**Figure 1:**
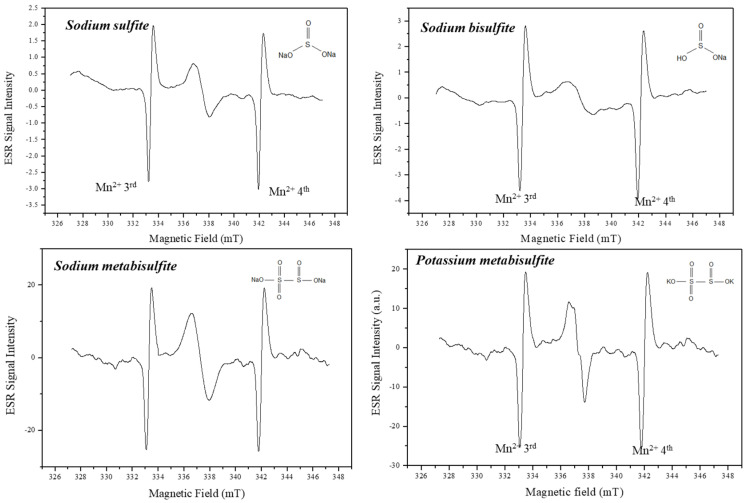
ESR signal of non-irradiated sulfites and their chemical structure. Spectra were recorded with Mn marker.

Figure 1 shows the ESR spectra of samples before irradiation. The background signal may be due to intrinsic defects present in the samples, which were commercial salts and used as received without any further treatment to simulate a practical situation, and can be used as a fortuitous dosimeter. The spectra of sulfites are shown with the Mn^2+^ marker that was used for determining the g-factor. The radicals present in sodium sulfite, sodium bisulfite and sodium metabisulfite are characterized by a relatively simple ESR spectrum, with the g-factor around g = 2.0085 and a width of 0.4 mT. Potassium metabisulfite presents an asymmetric line leading to the hypothesis of the presence of two or more radicals or crystal orientation in relation to the magnetic field. 

**Figure 2:**
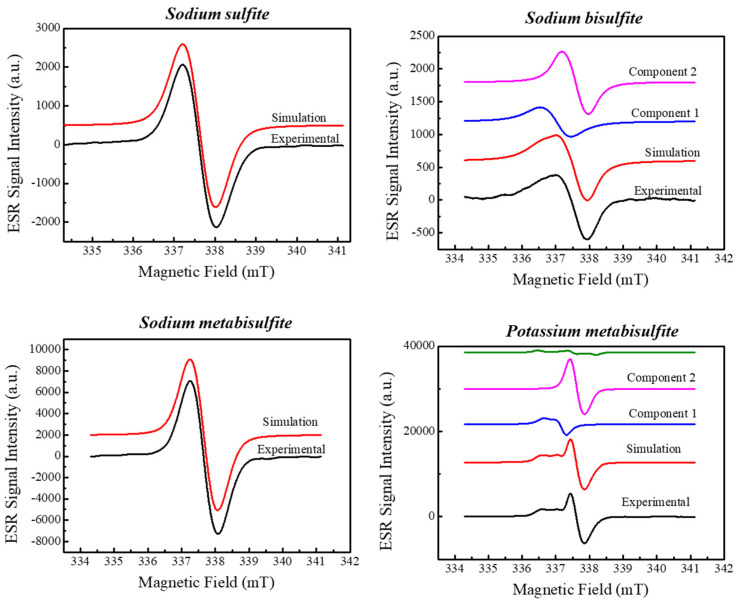
Simulation of ESR spectra of sulfites irradiated with 500 Gy.

As already mentioned, potassium metabisulfite appears to have a composite line structure, different from the other sulfites studied. Thus, Figure 2 shows the simulation (EasySpin [13]) of the sulfites studied after irradiation with a dose of 500 Gy for comparison. All radicals identified with 500 Gy fitted in the lower dose spectra. Table 1 lists the parameters found in the spectra simulation.

Despite the spectral appearance of isotropic lines of the radical of sodium sulfite, sodium bisulfite and sodium metabisulfite, the spectral simulation details orthorhombic symmetry. A similar result is reported by Gustafsson et al. [14] that found axial symmetry for an SO_3_^−^ radical in irradiated potassium dithionite. On the other hand, Chanty et al. [15] describe an isotropic line for an SO_3_^−^ radical in irradiated sodium dithionite, which leads us to conclude that although the nature of the radical is the same, its spectrum depends on the compound in which it is present.

**Figure 3:**
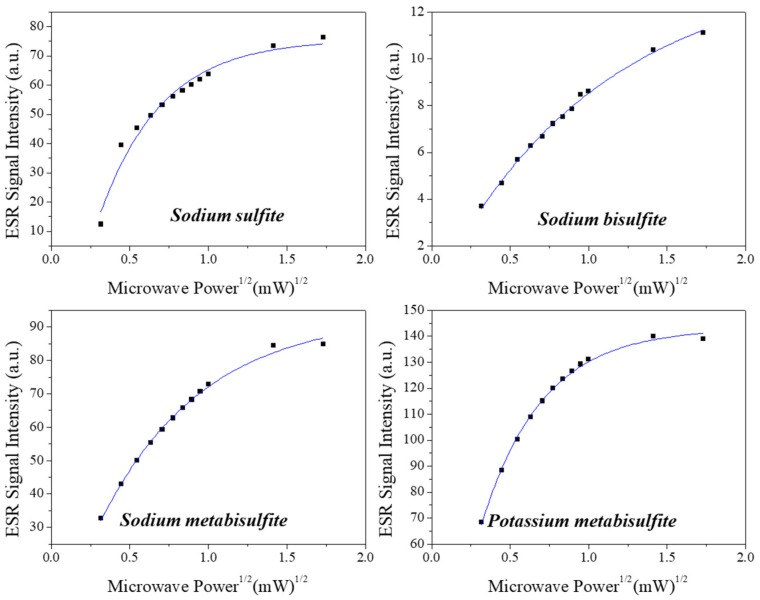
ESR signal amplitude as a function of the microwave incident power at the resonant cavity.

The power saturation curve is presented in Figure 3, considering the peak-to-peak amplitude of the ESR first derivative absorption line normalized by sample mass.

The signal intensity as a function of microwave power for all sulfites demonstrates a conventional characteristic, with a signal that increases with power^1/2^, with subsequent saturation of the signal up to 3 mW, which is a usual characteristic of spin–spin relaxation. The figure shows fitting with a single saturation exponential curve. For comparison purposes, samples with the same dose (500 Gy) were used with the signal normalized by mass. Sodium Bisulfite presented the smallest value of signal intensity at saturation and potassium metabisulfite reached the highest. 

**Figure 4:**
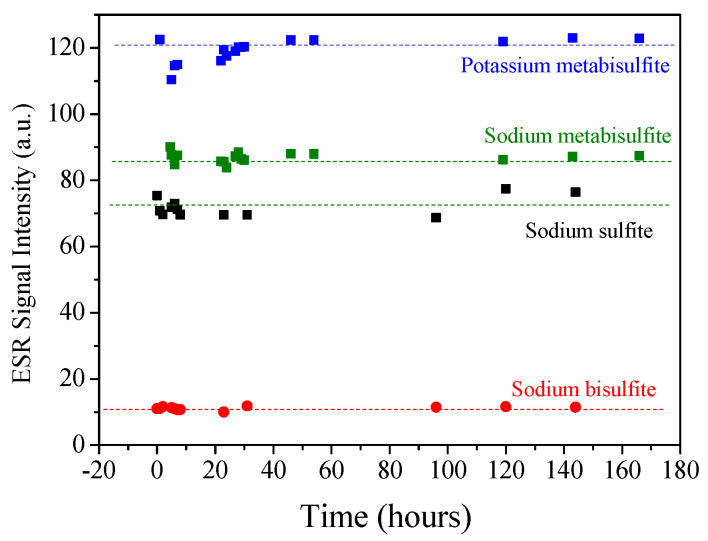
ESR signal amplitude of sample irradiated with a dose of 500 Gy as function of time after irradiation, showing the stability of signals during this period.

The stability of the ESR signal is crucial for retrospective dosimetry. So, the signal intensity as a function of time after irradiation was monitored until 166 h. Figure 4 shows the results, all samples were irradiated with 500 Gy, and the signal was normalized by sample mass for comparison. During this period of study, there was no fading of signals. As already mentioned, the high signal produced with a dose of 500 Gy was valuable as it gave better precision in determining the fading and the microwave power saturation.

### 2.2. Dose-Response Curve

Table 2 summarizes the best spectrometer parameters for ESR dosimetry with the materials. The modulation amplitude was selected to optimize the signal without its distortion. For sodium sulfite, bisulfite and metabisulfite, a relatively large modulation was employed to increase the signal amplitude. This procedure has been used in other studies [16,17] and showed an alternative when the signal intensity must be correlated with some variable, such as the radiation dose in the present case. 

**Figure 5:**
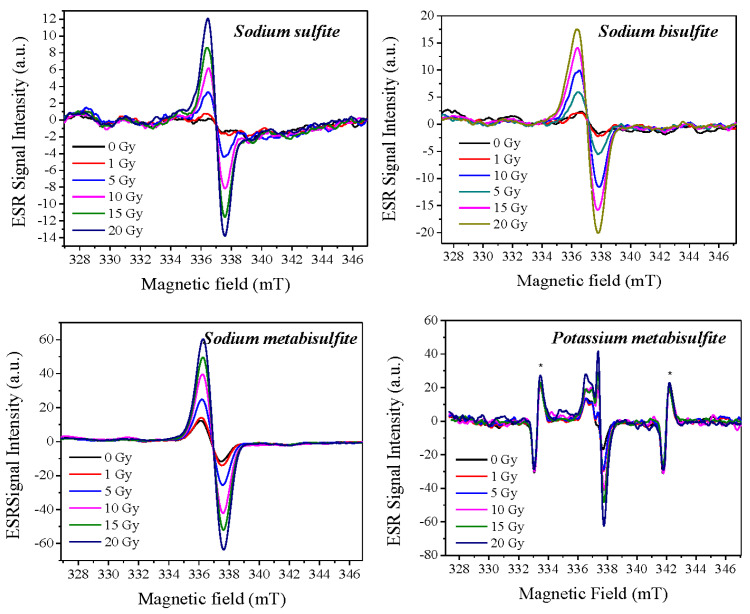
Spectra of sulfites corresponding to each absorbed dose to build the Dose–Response Curve. The 3rd and 4th Mn^2+^ lines of marker are indicated in the Potassium metabisulfite figure (*).

The ESR spectra of the sulfites as a function of the dose are shown in Figure 5. We can observe that the background signal, already present in the materials, has the same structure as the signals induced by ionizing radiation. The amplitude of the background signal was computed in the construction of the Dose–Response curve, ensuring that the calibration of the dosimeter is carried out taking this character into account. We can also notice that the signals increase in intensity with the radiation dose, and, in this dose range, no other species appear. Further experiments are needed, and future work can be performed with DFT to simulate the spectra to identify the nature of the radicals. Three of the compounds exhibit mostly a single ESR line but potassium metabisulfite shows a composite spectrum as demonstrated in the spectral simulation (Figure 2). Thus, the peak-to-peak of the main line was used to construct the Dose–Response curve and a smaller value of field modulation, in comparison to the other compounds, was employed. 

**Figure 6:**
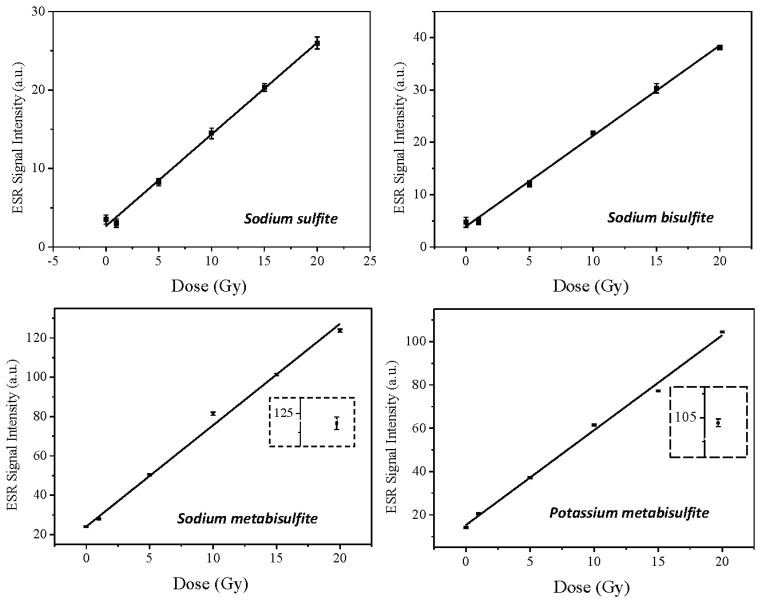
Dose–Response curves of sulfites studied. Linear fitting was used to simplify the data fitting. The inset shows the error box associated with the highest dose for the metabisulfite plots.

Most substances used for ESR dosimetry exhibit exponential behavior over a wide range of doses, up to kGy (Equation (1)). However, for the low dose range, as in the present paper, linear adjustment can be applied, as it is compatible with the beginning of the exponential curve (Equation (2)). Therefore, the experimental data points (Figure 6) were adjusted by linear fitting (Equation (2)), and Table 3 summarizes the parameters of fitting for each sulfite.

(1) $$I=I_{0}\times \left[1-e^{-\left(\frac{D+\alpha }{D_{0}}\right)}\right]$$

(2) $$I=I_{0}\times \left(1+\frac{D}{\alpha }\right)$$

where **I** is the signal intensity; **D**, the dose; **I_0_**, the linear coefficient in Equation (2) and Intensity at saturation in Equation (1); **α,** the dose related to **I** = 0; and **D_0_**, the dose at saturation. Table 3 summarizes the parameters of fitting for each sulfite. 

The Dose–Response curves obtained with optimized acquisition parameters for each compound studied demonstrate that metabisulfites are more sensitive to irradiation, because the curves, for the same dose range, reach higher intensities in relation to sodium sulfite and sodium bisulfite. This can be explained by observing that chemical structures composed of more elements show greater intensity of signal, due to the larger quantity of free radicals and their stability. Additionally, tests with radiation doses greater than 20 Gy may confirm the linear behavior of dose–response curves, so that sulfites can be used for high-dose dosimetry.

Therefore, these characteristics open space for future research, in which the behavior can be studied in relation to higher dose ranges, as well as to other energy ranges, opening perspectives for their applications. 

## 3. Materials and Methods

Potassium metabisulfite (Synth), sodium bisulfite (Sigma-Aldrich), sodium metabisulfite (Reagen) and sodium sulfite (Nuclear) of analytical grade were all commercially obtained in powder form and used as received. The samples were not crushed or sieved to avoid creation of defects by mechanical action. 

### 3.1. Irradiation

For irradiation, each material was placed in a small capsule and positioned between a solid water slab with thickness of 1.5 cm on top, corresponding to the build-up region for 6 MV energy, and a 15 cm solid water slab below, to allow proper back scattering conditions. The irradiation was performed with a Siemens Mevatron 6 MV clinical linear accelerator (Linac) with dose values varying from 1 to 20 Gy, for dose response investigation. This dose range was chosen with the aim of using these compounds as fortuitous dosimeters. The samples were positioned at source surface distance (SSD) of 100 cm at the Linac isocenter and irradiated with a 2 Gy/min dose rate in a 10 × 10 cm^2^ square field size. An aliquot of each material was irradiated with a dose of 500 Gy through Cesio-137 source for determination of the spectrum components by computer simulation, signal stability experiments and characterization in relation to microwave power. Such a high dose was employed to fully reveal all possible radicals created by ionizing radiation allowing a more precise spectral simulation and identification of all the radicals. 

### 3.2. ESR Spectra Characterization

The spectra were recorded in a JEOL–JES FA 200 X-band spectrometer, with cylindrical resonator, mode TE_011_. A Mn^2+^ marker was recorded simultaneously with the sample allowing the determination of the g-factor of each sulfite with JEOL software.

The sample was placed in 4 mm diameter high-purity quartz tubes for ESR measurements. Initially, the spectra of the sample before and after irradiation with 500 Gy were recorded for spectral characterization such as determination of the g-factors of radicals. Then, the signal intensity, considered as the peak-to-peak amplitude of the main line obtained by the first derivative of the absorption spectrum normalized by the mass quantity, was monitored as function of microwave power. The value of power that resulted in the higher intensity, without signal saturation, was adopted to construct the Dose–Response curve. Moreover, the signal was monitored over time to study its stability.

To confirm the assignment of the induced radicals by radiation, spectra simulation with the software EasySpin [13] was compared with experimental results. 

### 3.3. Dose-Response Curve

For Dose-Response Curve construction, the peak-to-peak amplitude of the main signal normalized by sample mass as function of dose was used. The software Origin (OriginLab) was used to adjust the experimental data with the linear or exponential function. 

## 4. Conclusions

Sulfites studied in this work presented a proportional response with radiation and stable ESR signal, enabling their use as a dosimeter. The materials used in this study could be good candidates for accidental dosimetry studies since their presence in many food and pharmaceutical factories makes their use possible in the case of a radiological accident. Further studies are required to determine the lowest detectable dose, identification of radicals and influence of the energy of the radiation source among others, to establish a complete protocol for their use.

## Figures and Tables

**Table 1:** Parameters of Spin Hamiltonian of sulfites, g-factor and linewidth (LW).

Compound	g-Factor	LW (mT)
Sodium sulfite	2.0085 2.0062 2.0062	0.6266 0.2427
		
Sodium bisulfite	2.0133 2.0089 2.0098	0.3168 0.6736
	2.0047 2.0071 2.0098	0.0083 0.3740
		
Sodium metabisulfite	2.0089 2.0045 2.0067	0.5546 0.2112
		
Potassium metabisulfite	2.0130 2.0093 2.0092	0.9715 0.2011
	2.0052 2.0074 2.0078	0.2404 0.0961

**Table 2:** X-band ESR spectrometer settings for Dose–Response Curve.

Parameter	Sodium Sulfite	Sodium Bisulfite	Sodium Metabisulfite	Potassium Metabisulfite
Center magnetic field	~337 mT			
Microwave frequency	9 GHz			
Resonant Cavity	Cylindrical resonator, mode TE011, Q factor 6000			
Modulation frequency	100 kHz			
Microwave power	1 mW			
Modulation amplitude	1.0 mT	1.4 mT	1.4 mT	0.1 mT
Sweep width	10 mT			
Time constant	0.3 s			
Gain	10 × 100			
Sweep time	1 min			
Number of scans	3			
Sample mass	~80 mg	~85 mg	~60 mg	~90 mg

**Table 3:** Parameters of fitting of Dose–Response Curve for each compound studied.

Compound	Model	I_0_	α	Adj. R-Square
Sodium sulfite	linear	2.7	2.3	0.997
Sodium bisulfite	linear	4.0	2.3	0.997
Sodium metabisulfite	linear	24.1	4.7	0.997
Potassium metabisulfite	linear	15.3	3.5	0.995

## Data Availability

The data can be made available by the authors upon request.

## Sample Availability

Samples of the all compounds studied in this paper are available from the authors.
